# Transient and sustained cortical activity elicited by connected speech of varying intelligibility

**DOI:** 10.1186/1471-2202-13-157

**Published:** 2012-12-31

**Authors:** Hannu Tiitinen, Ismo Miettinen, Paavo Alku, Patrick J C May

**Affiliations:** 1 Department of Biomedical Engineering and Computational Science, Brain and Mind Laboratory, Aalto University School of Science, Espoo, Finland; 2 Department of Signal Processing and Acoustics, Aalto University School of Electrical Engineering, Espoo, Finland; 3 BioMag Laboratory, HUS Medical Imaging Center, Helsinki University Central Hospital, Helsinki, Finland

**Keywords:** Acoustic distortion, Auditory evoked magnetic fields, Auditory cortex, Human, Intelligibility, Magnetoencephalography, N1m, P2m, Speech processing, Sustained field

## Abstract

**Background:**

The robustness of speech perception in the face of acoustic variation is founded on the ability of the auditory system to integrate the acoustic features of speech and to segregate them from background noise. This auditory scene analysis process is facilitated by top-down mechanisms, such as recognition memory for speech content. However, the cortical processes underlying these facilitatory mechanisms remain unclear. The present magnetoencephalography (MEG) study examined how the activity of auditory cortical areas is modulated by acoustic degradation and intelligibility of connected speech. The experimental design allowed for the comparison of cortical activity patterns elicited by acoustically identical stimuli which were perceived as either intelligible or unintelligible.

**Results:**

In the experiment, a set of sentences was presented to the subject in distorted, undistorted, and again in distorted form. The intervening exposure to undistorted versions of sentences rendered the initially unintelligible, distorted sentences intelligible, as evidenced by an increase from 30% to 80% in the proportion of sentences reported as intelligible. These perceptual changes were reflected in the activity of the auditory cortex, with the auditory N1m response (~100 ms) being more prominent for the distorted stimuli than for the intact ones. In the time range of auditory P2m response (>200 ms), auditory cortex as well as regions anterior and posterior to this area generated a stronger response to sentences which were intelligible than unintelligible. During the sustained field (>300 ms), stronger activity was elicited by degraded stimuli in auditory cortex and by intelligible sentences in areas posterior to auditory cortex.

**Conclusions:**

The current findings suggest that the auditory system comprises bottom-up and top-down processes which are reflected in transient and sustained brain activity. It appears that analysis of acoustic features occurs during the first 100 ms, and sensitivity to speech intelligibility emerges in auditory cortex and surrounding areas from 200 ms onwards. The two processes are intertwined, with the activity of auditory cortical areas being modulated by top-down processes related to memory traces of speech and supporting speech intelligibility.

## Background

Successful comprehension of connected speech can be seen as an auditory scene analysis problem [1] involving the matching of the acoustic properties of the incoming voice signal with memory representations of speech. This is not a linear bottom-up process, but one that can be modified in a top-down fashion by long-term memory traces of, for example, one’s native language mediating syntactic and semantic content [2,3] as well as information concerning affective aspects of the speaker [4]. Prior expectations of the stimuli can dramatically alter the perception of acoustically distorted speech, rendering initially unintelligible stimuli entirely comprehensible (see e.g. [5]). Despite increasing efforts in the study of the neural basis of speech perception, it has proven difficult to distinguish between the cortical processes related to the bottom-up extraction of acoustic features of speech and facilitating top-down mechanisms, such as recognition memory for linguistic content. One reason for this is that in most studies the effects related to speech comprehension have been investigated by comparing brain responses to intact speech with those elicited by acoustically degraded versions of the stimuli. Having two or more acoustically different types of stimuli poses a major problem: Acoustic variability in itself leads to differences in the response and, consequently, the relative contributions of acoustic feature processing and speech comprehension become confounded and, thus, it is difficult to isolate the effects specific to speech comprehension. An experimental paradigm which controls for acoustic variability would appear to be needed in order to simultaneously capture both the bottom-up and top-down aspects of speech comprehension.

In electro- and magnetoencephalography (EEG & MEG, respectively) recordings of the human brain, the presentation of short-duration (<200 ms) auditory stimuli typically results in transient responses and, in the case of long-duration stimuli (>300 ms), the transient responses are followed by a sustained response. These responses are suited for revealing both the spatial and temporal evolution of cortical activity. Notably, the transient auditory N1m response generated in the auditory cortex [6] is sensitive to multiple aspects of speech such as fundamental frequency [7], formant transitions [8], intonation [9], place of articulation [10,11], the periodic structure of vowel sounds [12-15], and the phonetic features of consonants [16]. These observations indicate that the auditory cortex carries out parallel bottom-up processing of the acoustic properties of speech sounds, independently of the subject’s attentional focus. Further, the N1m has also been found to be a sensitive measure of the extraction process with which the human brain segregates speech signals from various types of noise contributions [17-19]. While these EEG/MEG studies, utilizing short (~200 ms) isolated vowel sounds in no-task (passive) recording conditions, have revealed the link between transient activation and the bottom-up extraction mode in acoustic feature processing, the role of top-down influences on the processing of meaningful speech has remained largely unaddressed. Therefore, the above studies should be complemented by investigations focusing on the sustained activity elicited by connected speech, thus potentially revealing how activity spreads to multiple cortical brain areas performing speech processing.

A growing body of hemodynamic evidence suggests that the cortical processes underlying speech comprehension operate in a hierarchical fashion, with the auditory cortex activated by the acoustic properties of sounds (see, e.g. [20]), and the regions anterior and posterior to auditory cortex being sensitive to the intelligibility of speech [21-28]. The anatomical areas underlying the speech comprehension network have been investigated by contrasting cortical responses to speech with responses to closely matched non-speech stimuli, such as noise-vocoded sounds [21,23-26] or tonal stimuli [27,29]. These studies indicate that areas in the superior temporal sulcus (STS) respond more vigorously to speech than to non-speech stimuli, and that STS regions anterior and posterior to auditory cortex are sensitive to the intelligibility of the stimuli. In addition, when speech stimuli with different levels of intelligibility are presented, overlapping temporal cortical areas seem to be activated during passive listening [26], active listening [23,24], and active recognition tasks [21,25,28].

Currently, the evidence pertaining to the role of auditory cortex seems to be contradictory, with findings indicating that this brain region is either sensitive [30] or insensitive to speech intelligibility (e.g. [22,25]). Given that top-down information - such as prior expectations of the stimuli - can substantially alter the perception of degraded speech [5], it seems plausible that the extraction of acoustic cues from the distorted signal might be enhanced through feedback connections from higher-order cortical areas to auditory cortex. As a result, these changes in cortical processing might be observed in the activity of the auditory cortex as well. Given that hemodynamic measures lack in temporal acuity, the millisecond resolution of EEG/MEG measures of transient and sustained brain activity (presumably confounded in hemodynamic measurements) might provide complementary information to fMRI findings on this issue.

The current MEG study assesses how the intelligibility of connected speech is reflected in the temporal evolution of activity in auditory cortex and surrounding areas. The study capitalizes on the phenomenon that the intelligibility of speech signals can be manipulated without changing the acoustic structure of the stimulus. Accordingly, the experiment consisted of three consecutive sessions during which the same set of sentences was presented in distorted, undistorted, and - once again - in distorted form. As a result, acoustically *identical* distorted stimuli were perceived as either unintelligible or intelligible, depending on whether the subject had previously been exposed to an undistorted, intact version of the sentence. Any possible change in brain activity, then, cannot be attributed to changes in the acoustic structure of the stimuli but, rather, to the top-down processes related to speech comprehension. We hypothesized that the activity generated in auditory cortex and reflected in the N1m and P2m responses would be sensitive to the acoustic variation in the speech signal [17-19] whereas the sustained activity following transient activity might be responsive to whether speech is intelligible, and less sensitive to the acoustic attributes of the stimuli. Given the novelty of the proposed experimental paradigm, our aim was to proceed with caution and to provide a tentative description of brain events related to speech intelligibility uncontaminated by the effects caused by attentional engagement (arousal level, sustained attention, etc.) as well as by planning and execution of motor responses. Therefore, the current study focuses on brain activity obtained in the passive recording condition.

## Methods

### Participants

Ten healthy, right-handed volunteers (age range 19-28 years; mean=22.0; SD=2.93) participated in the study with written informed consent. In a pre-measurement questionnaire, all the participants declared themselves to be native, right-handed Finnish speakers with normal hearing. The experiments were approved by the Ethical Committee of the Helsinki University Central Hospital.

### Stimulus material

The stimuli were created from speech data spoken in Finnish by a professional logopaedist. The recordings were made in an anechoic chamber with a high-quality condenser microphone (Bruel&Kjaer 4188). The speech waveforms were sampled at a rate of 22.05 kHz with an amplitude resolution of 16 bits. To remove any low-frequency fluctuation picked up by the microphone, the signals were high-pass filtered with a 6th order Butterworth filter (cut-off frequency at 60 Hz).

The speech data recorded consisted of 84 Finnish sentences, comprising six to seven words (3-4.4 s in duration) of the Finnish language. The sentences were constructed from three parts, including seven starting words, three sentence stubs, and four ending words. The starting and ending word was always a noun whereas sentence stubs involved nouns, objectives and verbs. In order to prevent any transient, time-locked activity occurring in the averaged data during the sustained field time range (300-3000 ms), the sentence stubs were constructed so that the acoustic structure of all the sentence stubs deviated from each other. This procedure resulted in a set of syntactically correct sentences which included both semantically meaningful (e.g. “The news broke out that a street was built into the village”) as well as meaningless (e.g. “The news looked as strange as a bottled street”) exemplars. In order to arrive at a sufficient signal-to-noise ratio (SNR) in MEG recordings, each subject was presented with a total of 120 sentences, with 36 random repetitions from the original 84-sentence set.

The quality of the generated sentences was degraded by decreasing the amplitude resolution of the temporal waveform using the uniform scalar quantization (USQ) technique [31]. In this procedure, the maximum absolute value of each sentence is first determined. By using this value, the sentence is scaled to cover the full dynamics of the 16-bit amplitude scale, that is, each signal sample is rounded off to its nearest integer number and there are altogether 65536 such integers between the smallest negative value (-32768) and the largest positive value (32767). A distorted version of each sentence is then computed by using 1-bit quantization, in which the number of quantization levels is radically reduced from 65536 to just two. All in all, this quantization procedure yielded two versions of each sentence which, in the following, will be referred to as the undistorted (16-bit) and distorted (1-bit) sentence. After the quantization, the intensity (in terms of square sum of time-domain signal values) of the undistorted and distorted sentence was equalized and, finally, the onsets and offsets of the stimuli were smoothed with a 5-ms Hann window. Examples of waveforms can be found in [17-19].

### Experimental design

**Figure 1:**
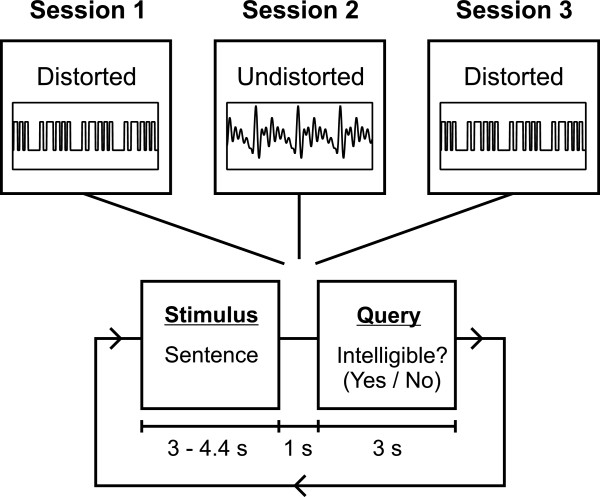
**Experimental design.** The experiment consisted of three sessions in which the subjects were presented with acoustically distorted sentences (Session 1), followed by undistorted sentences (Session 2), after which the distorted sentences were presented once again (Session 3). After each sentence, the subjects indicated whether they had understood the sentence by pressing a Yes/No response key.

The experiment was designed to study the effects of acoustic degradation and intelligibility of connected speech on cortical activity (Figure 1). During the experiment, the participants first listened to the distorted sentences (Session 1), then to the undistorted versions of these sentences (Session 2), and finally to the distorted sentences again (Session 3). The offset-to-onset inter-sentence interval was 4 seconds. The subjects were instructed to focus their gaze on a fixation cross while listening. The sentence was followed by a 1-sec break. Subsequently, a question screen inquiring whether the sentence was intelligible or not was displayed, and the subject responded with a button press (Yes/No forced-choice task) within a 3-sec time window. After the active recording condition, the sentences were presented in the passive condition during which the subjects were under instruction to watch a silent movie while ignoring the auditory stimuli.

Intelligibility of noise-distorted speech has been studied widely in psychoacoustics and speech communication technology, and both objective and subjective assessment measures of speech intelligibility have been adopted. Objective measures to predict speech intelligibility in the presence of noise involve, for example, the articulation index [32], the speech transmission index [33], and the speech intelligibility index [34]. Subjective measures make use of listeners and various types of speech material, either synthetic or natural, involving stimuli such as phones, words, sentences and sometimes even free conversation. In subjective evaluation, speech stimuli are typically played only once to listeners who then write down what they believe to have understood when listening to the corrupted utterances. An intelligibility score is then computed as a percentage of speech elements correctly reported. Subjective intelligibility scores used in speech perception studies include, for example, word error rates [35] as well as sentence and consonant identification rates [36]. In the current study, however, neither objective measures nor the subjective scoring methods mentioned above were used in the evaluation of speech intelligibility. Instead, subjective intelligibility was evaluated by asking the subject to grade the speech sentence in a binary manner as either completely intelligible or unintelligible. By choosing the former, the subject indicated that she/he had understood the sentence correctly. By choosing the latter, the subject indicated that she/he was unable to understand the sentence. Instead of counting quantitatively the percentage of words correctly recognized, this binary subjective intelligibility score measures solely whether the subject considers that she/he was able to understand the meaning of the heard sentence. Therefore, it is a genuinely subjective means to evaluate intelligibility of corrupted speech and well suited for use in the present study to investigate speech processing in human subjects.

### Data acquisition

Auditory evoked fields were recorded using a 306-sensor whole-head MEG device (Vectorview 4-D, Neuromag Oy, Finland) in a magnetically shielded room. The recorded signals obtained by the 204 gradiometers were sampled at a rate of 0.6 kHz and low-pass filtered online with a cut-off frequency of 172 Hz. Horizontal and vertical eye movements were monitored by two electrode pairs. The position of the participant’s head with respect to the MEG sensor array was determined with four head-position indicator (HPI) coils before the beginning of each measurement session. The HPI coils were localized with respect to the nasion and the preauricular points using a 3-D digitizer. The head-based coordinate system was defined by the *x*-axis passing through the preauricular points (positive to the right), the *y*-axis passing through the nasion (positive to the front), and the *z*-axis as the vector cross product of the *x*- and *y*-unit vectors. The participant was instructed to remain stationary during the experiment. The auditory stimuli were binaurally delivered to the subject’s ears through plastic tube earphones with an average intensity of 65 dB SPL.

### Offline preprocessing of the MEG data

In order to exclude gradiometer sensors with recording artifacts or a low SNR, the recorded data was first visually inspected. To suppress magnetic noise, spatio-temporal signal-space separation (Maxwell filtering with temporal extension) was performed for the data using Elekta Neuromag MaxFilter. Two data sets with different filtering and averaging parameters were extracted from the data, one for examining the transient N1m and P2m responses, and the other for studying the sustained field (SF). The data set for the transient response analysis was filtered with a 2-30 Hz band-pass filter, and the data set for the sustained field analysis with a 30 Hz low-pass filter.

For averaging the data, the epochs were time-locked to the beginning of the stimuli, and amplitude-corrected using a 100-ms pre-stimulus baseline. Epochs with excessive magnetic field gradient amplitudes (over 2000 fT/cm) or with large eye movements (electro-oculogram threshold = 150 μV) were excluded from the average. A 500-ms window was used in averaging the transient response data, and a 3000-ms window for the sustained field data. The data of one subject was discarded due to a weak SNR and eye-movement artifacts. Furthermore, the data of another subject was excluded from the analyses of the sustained field due to an insufficient number of averaged epochs.

### MEG data analysis

#### Latencies, amplitudes and source localization of the transient responses

The latencies and amplitudes of the N1m and P2m responses were determined separately in each hemisphere from the peak values of the responses. The peak amplitude was calculated as the maximum magnitude of the vector sum from the sensor pair exhibiting the maximum response within a 90-140 ms time window for the N1m, and a 150-250 ms window for the P2m response.

The source locations of the N1m and P2m responses were estimated by equivalent current dipole (ECD) analyses [37] conducted separately in the left and right hemisphere. A single ECD was fitted to the data at the N1m and P2m peak latency using a subset of 44 gradiometer sensors covering the temporal areas of each hemisphere. A spherical model was used to model the conductivity of the head. The ECD analyses were carried out using the Elekta Neuromag Xfit Source Modeling Software.

#### Amplitude of the sustained field

The sensor pairs yielding the maximal transient responses also exhibited prominent sustained fields. The amplitude of the sustained field (SF) was quantified as the mean amplitude of the vector sum over a predefined time interval. Two distinct phases were observed in the AEF waveform: a large deflection within the 300-1000 ms time window, and a relatively static part within the 1000-3000 ms time interval. Thus, in each hemisphere, the amplitude of the sustained field was calculated separately over the 300-1000 ms (early SF), and 1000-3000 ms (late SF) time intervals.

#### Current distribution estimates

To study the spatial distribution of cortical activity, noise-normalized minimum-norm estimates (MNEs) were calculated. To this end, noise-covariance matrices were computed from the 100-ms pre-stimulus baselines of the individual epochs in the Maxwell-filtered raw data. The forward solutions and the inverse operators were calculated for each session by employing a boundary-element model computed using average head and skull surface reconstructions provided with the MNE-Suite Software.

MNE and sLORETA [38] estimates were computed at 5-ms intervals for the data of each individual subject. To study the current distribution during the transient responses, both estimates were averaged over 40-ms time windows centered at the peaks of the N1m and P2m responses. The peak latencies of the responses were obtained from the gradiometer analyses (see above). Fixed time windows of 300-1000 ms (early SF) and 1000-3000 ms (late SF) were used in analyzing the current distribution during the sustained field responses.

#### Region of interest analysis

**Figure 2:**
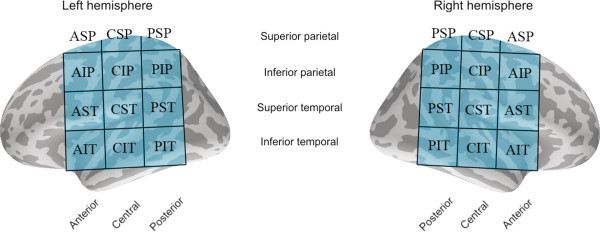
**Regions of interest (ROIs) used for calculating the mean currents in the MNE analyses.** The grid provides a rough parcellation of brain areas in the anterior/central/posterior, inferior/superior, and parietal/temporal dimensions.

The cortical surface used in calculating the MNE estimates was divided into 24 regions of interest (ROIs), 12 regions in each hemisphere. The ROIs used in the analyses are depicted in Figure 2. The ROIs were labeled according to their physical location: anterior/central/posterior, superior/inferior, and temporal/parietal region. The ROIs were selected from the parietal and temporal cortices with emphasis on examining the spread of activation from the primary auditory cortical areas within the superior temporal gyrus to regions anterior and posterior to the auditory cortex, and to the parietal regions [39,40]. The ROIs were centered on the auditory cortical areas within the superior temporal gyrus (CST in the current notation, see Figure 2). We emphasize that due to a lack of individual structural MRI data, the ROIs should be regarded as approximations of particular cortical areas only. For example, Wernicke’s and Broca’s area lie roughly within PST and AST, respectively, and motor areas within the pre- and postcentral gyrus can be found in AIP and CIP, respectively [41]. The mean current for each ROI was calculated separately for the transient and sustained responses as the average of the MNEs over all the voxels within each ROI (see previous subsection; see also [42]). The MNE values were extracted from the original MNEs without noise normalization.

### Statistical analyses

Repeated-measures analysis of variance (ANOVA) was used to analyze 1) the amplitudes, latencies, and source location of the transient responses, 2) the mean amplitudes of the sustained responses, and 3) the mean currents within each ROI. All the ANOVAs were of the design 2×2×3, with the within-subjects factors of hemisphere (left/right), recording condition (active/passive), and degradation (distorted first presentation/undistorted/distorted second presentation). The effects of intelligibility (based on the current behavioral results) on the cortical activity measures were analyzed by post-hoc comparisons (Newman-Keuls test) of the degradation factor levels.

## Results

### Intelligibility of the sentences

**Figure 3:**
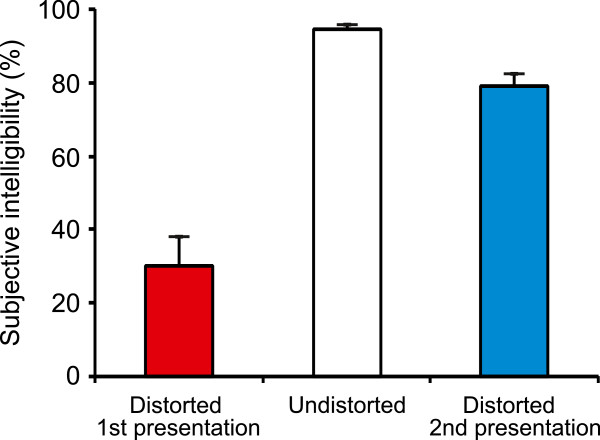
**Behavioral results.** In the case of the first presentation of the distorted sentences, the stimuli were very difficult to understand (mean subjective intelligibility rating = 30.2%). After an intervening presentation of the same sentences in undistorted form, the comprehensibility of the distorted sentences increased considerably (78.9%). Error bars indicate the standard error of the mean (SEM).

During the MEG measurements, the subjects first listened to sentences acoustically degraded through amplitude quantization. These were then followed by the same set of sentences in undistorted sound quality. Finally, the subjects heard the degraded sentences again (see Figure 1). Subjective intelligibility, that is, the proportion of sentences the subject reported having understood was 94.8% (±0.9%) for the undistorted sentences, and it increased substantially between the first and the second presentation of the degraded sentences for all of the subjects (Figure 3). The mean proportion of sentences reported as intelligible was 48.7 percentage points lower for the first presentation (30.2%; SEM: ±7.6%) than for the second presentation of the degraded sentences (78.9%; SEM: ±3.7%; *F*(1,7)=43.26, *p*<0.0005). This increase in subjective intelligibility from 30% to 80% for acoustically identical stimuli demonstrates how a single presentation of intact speech material can drastically alter the subject’s ability to comprehend degraded connected speech.

### Activity in auditory cortex

**Figure 4:**
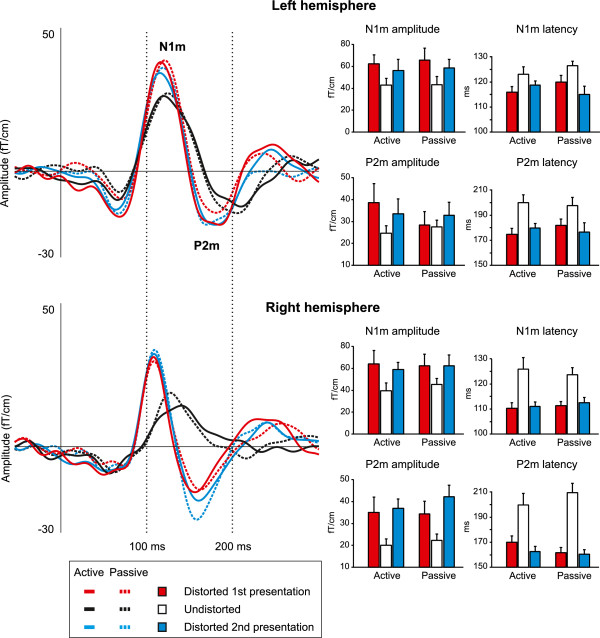
**Grand-averaged transient evoked fields measured from the left and right hemisphere and the amplitudes and latencies of the N1m and P2m responses.** The distorted sentences elicited N1m responses with a larger amplitude and an earlier peak latency than their undistorted counterparts. The N1m responses peaked later in the left hemisphere than in the right. The P2m responses were more prominent and occurred earlier for the distorted sentences than for their undistorted counterparts. Furthermore, the P2m latency and amplitude effects were more pronounced in the right hemisphere. The AEFs are from the sensor exhibiting maximum response, and the amplitude and latency results have been calculated from the vector sum from the sensor pair with the maximum response. Error bars indicate the standard error of the mean (SEM).

**Figure 5:**
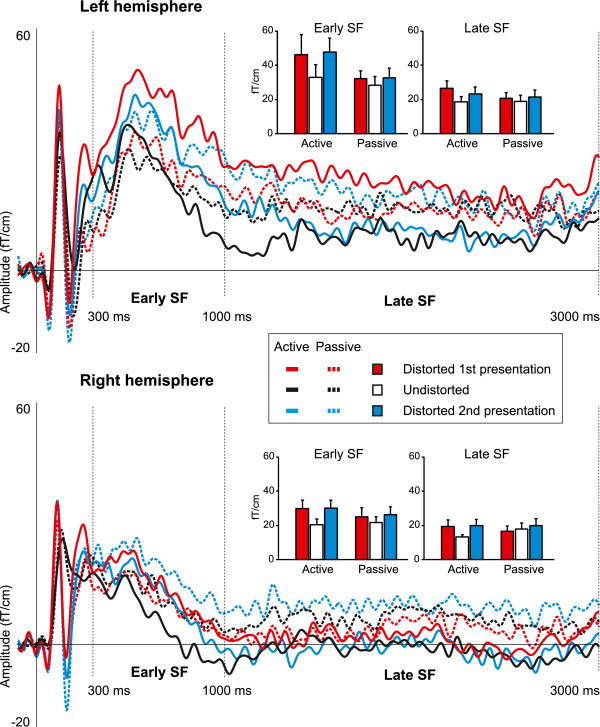
**Grand-averaged early and late phase of the sustained field (SF) measured from the left and right hemisphere.** As shown in the insets, the early SF (300-1000 ms) had a higher mean amplitude than the late SF (1000-3000 ms). Further, the early SF was larger in the active conditions than in the passive ones, and more prominent in the left hemisphere. The AEFs are from the sensor exhibiting maximum response, and the amplitude results have been calculated from the vector sum from the sensor pair with the maximum response. Error bars indicate the standard error of the mean (SEM).

In the first stage of the analyses, the local activation of the auditory cortex was studied separately in the left and right hemisphere by using the pair of gradiometer sensors in each hemisphere exhibiting the largest responses. Prominent N1m and P2m responses were elicited at the beginning of all sentences, as depicted in Figure 4, which shows the first 300 ms of the AEF. The AEF over the longer, 0-3000 ms period is shown in Figure 5.

#### Amplitudes of the transient responses

The mean amplitudes of the N1m and P2m responses were larger for the distorted sentences than for the undistorted sentences. The mean amplitude of the N1m increased from 42.8 fT/cm to 63.6 fT/cm as a result of sound degradation (Figure 4, *F*(2,16)=9.63, *p*<0.002). The P2m response was also larger for the distorted (33.9 fT/cm) than for the undistorted (22.1 fT/cm) sentences (Figure 4, *F*(2,16)=7.51, *p*<0.01). The effect of distortion on the P2m amplitude was more pronounced in the right hemisphere than in the left (*F*(2,16)=8.05, *p*<0.005). Post-hoc tests revealed that this hemispheric asymmetry was due to a smaller P2m response for the undistorted sentences in the right hemisphere (19.1 fT/cm) than in the left (25.2 fT/cm; *p*<0.001). Furthermore, the N1m and P2m amplitudes were equally large for the first and second presentation of the degraded sentences, indicating that the increase in perceptual intelligibility (see Figure 3) was not reflected in the amplitudes of the transient responses.

#### Latencies of the transient responses

In comparison to responses elicited by the undistorted sentences, degradation of sound quality resulted in earlier N1m and P2m responses (Figure 4). The latency of the N1m decreased from 125 ms to 114 ms (*F*(2,16)=19.07, *p*<0.0001), and that of the P2m from 202 ms to 172 ms (*F*(2,16)=17.91, *p*<0.0001). In addition, the mean latencies of the N1m were longer in the left hemisphere (120 ms) than in the right (116 ms; *F*(2,16)=11.47, *p*<0.01). However, the effect of distortion on the P2m latency was larger in the right hemisphere than in the left (*F*(2,16)=7.04, *p*<0.005), with the degraded stimuli eliciting the P2m response 15 ms earlier in the right (163 ms) than in the left (178 ms) hemisphere (*p*<0.05 in all comparisons). Moreover, given that both the first and the second presentation of the distorted stimuli resulted in unvarying N1m and P2m latencies, it appears that the intelligibility of the degraded sentences does not affect the timing of transient brain activity.

#### Source locations of the transient responses

Changes in the source locations of the transient responses were investigated by fitting a single equivalent current dipole (ECD) at the response’s peak latency in each hemisphere. The source locations of the N1m and P2m responses were modified by sound degradation and attention. In the left hemisphere, the ECDs for the N1m response were located 4.6 mm medial for the distorted stimuli (*x*=-49.5 mm) compared to those for the undistorted stimuli (*x*=-54.0 mm; *F*(2,16)=4.57, *p*<0.05). In addition, the N1m sources were more superior during active listening than in the passive conditions (*F*(1,8)=6.54, *p*<0.05). However, the effect of attention on the N1m source location depended on sound degradation (*F*(2,16)=4.23, *p*<0.05). Post-hoc tests showed that the N1m ECDs were more superior in the active condition only during the first presentation of the distorted sentences (*p*<0.02).

Sound degradation resulted in a 8.6-mm shift of the P2m sources along the anterior-posterior axis, the sources of the P2m being more posterior for the distorted stimuli (*y*=4.9 mm) than for the undistorted stimuli (*y*=13.5 mm; *F*(2,16)=18.89, *p*<0.0001). Furthermore, the P2m ECDs were more medial in the right hemisphere during active listening (*x*=44.0 mm) than in the passive conditions (*x*=51.1 mm; *F*(1,8)=9.08, *p*<0.02). The effect of attention on the lateral-medial position of the P2m sources was dependent on sound degradation (*F*(2,16)=4.19, *p*<0.05). More specifically, the ECDs for the active and passive conditions diverged only when the stimuli were undistorted (*p*<0.05).

#### Sustained responses

The early (300-1000 ms) and the late (1000-3000 ms) part of the sustained field (SF) were analyzed separately (Figure 5). The early SF had a higher mean amplitude than its late counterpart (20.4 – 47.8 fT/cm and 13.3 – 26.4 fT/cm for the early and late SF, respectively; *F*(1,7)=19.20, *p*<0.005). The early SF was stronger in the active conditions (34.6 fT/cm) than in the passive conditions (27.7 fT/cm; *F*(1,7)=7.81, *p*<0.05). In addition, the effect of sound degradation on the early SF amplitude approached statistical significance (*F*(1,7)=3.67, *p<*0.052), the distorted sentences yielding larger responses (33.4 fT/cm) than the undistorted ones (25.8 fT/cm). Post-hoc comparisons revealed that the early SF amplitude elicited by the first presentation of the degraded sentences was significantly increased in comparison to the amplitude for the undistorted stimuli (*p<*0.05). Overall, the early SF was more pronounced in the left hemisphere, with this asymmetry approaching statistical significance (*F*(1,7)=5.05, *p*<0.059). All other statistical comparisons yielded non-significant results.

### Activity in auditory cortex and surrounding areas

**Figure 6:**
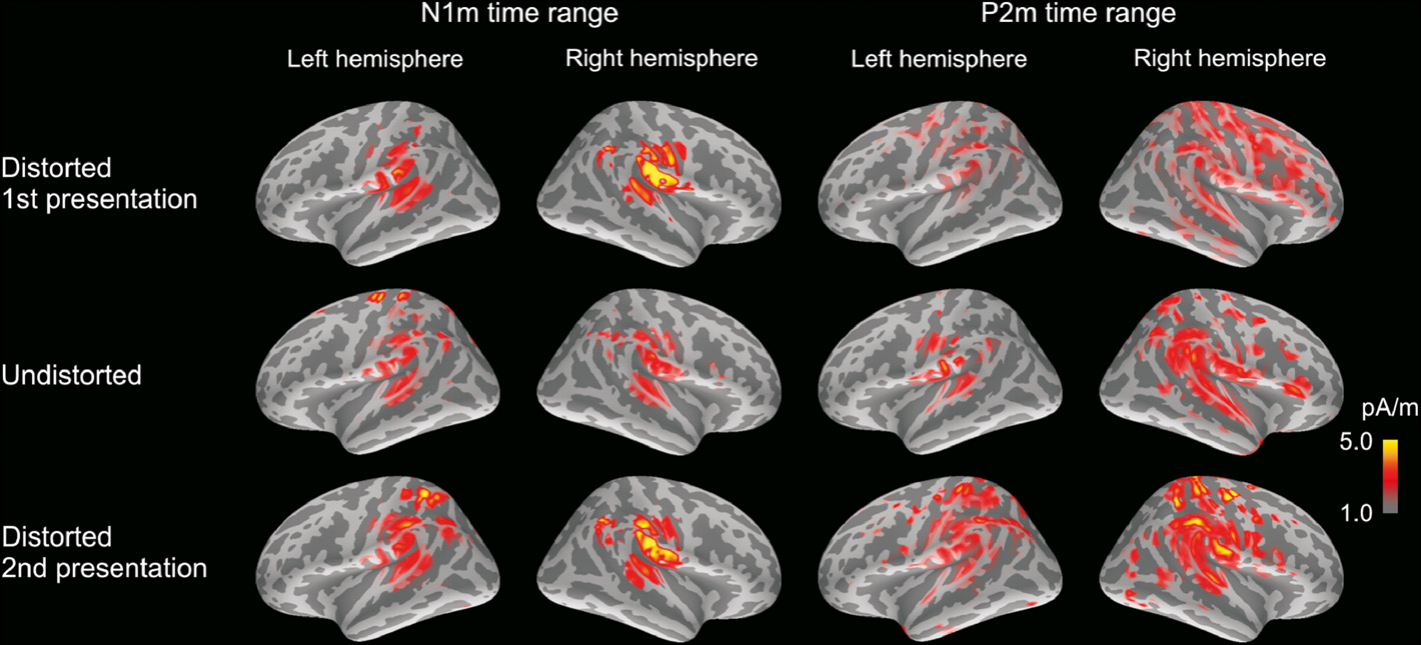
**Noise-normalized current distribution (MNE) in the left and right hemisphere during passive listening within the N1m and P2m time windows.** Both responses originated in the vicinity of auditory cortex, with the N1m being more focal than the P2m. Normalization of the MNE is with respect to the maximum value in the sLORETA estimate. Only the MNE voxels with a value over 50% of the maximum value in the sLORETA estimate are shown, and the same scaling is applied in all the estimates.

**Figure 7:**
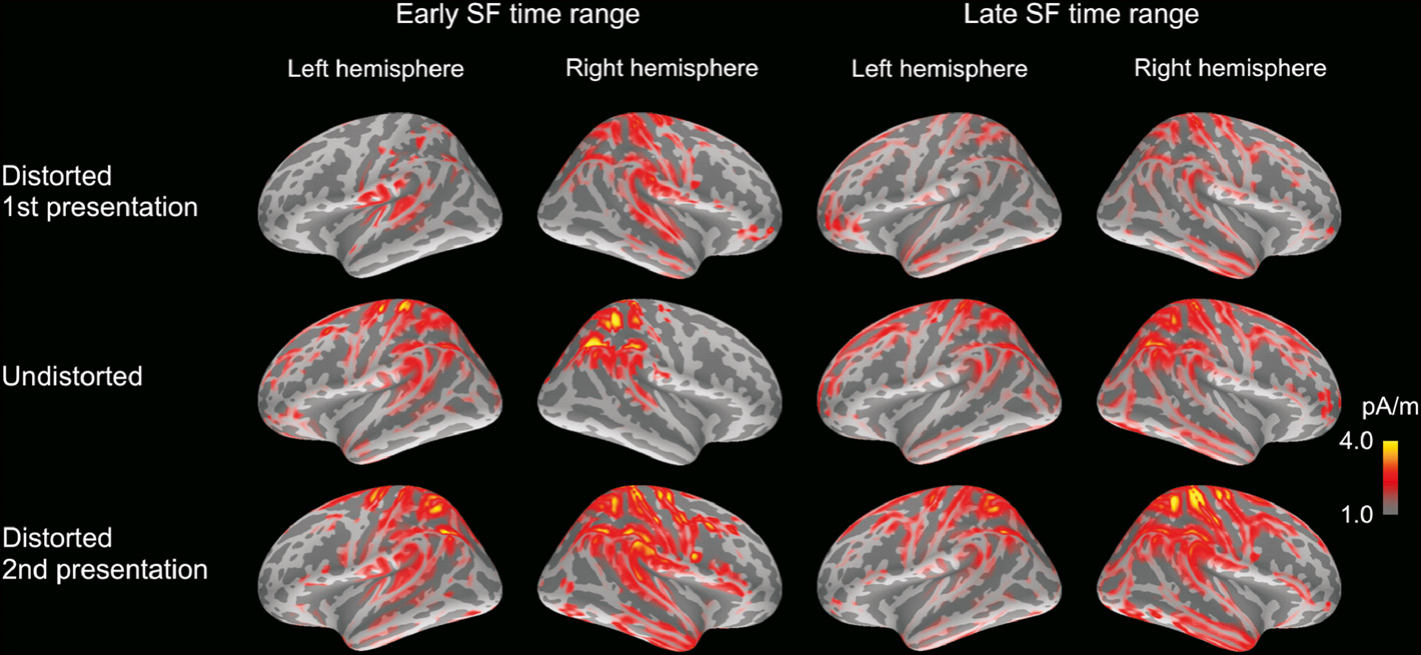
**Noise-normalized current distribution (MNE) in the left and right hemisphere during passive listening within the early and late SF time windows.** Activity in cortex was widespread during the generation of the early and late SF. Normalization of the MNE is with respect to the maximum value in the sLORETA estimate. Only the MNE voxels with a value over 50% of the maximum value in the sLORETA estimate are shown, and the same scaling is applied in all the estimates.

**Figure 8:**
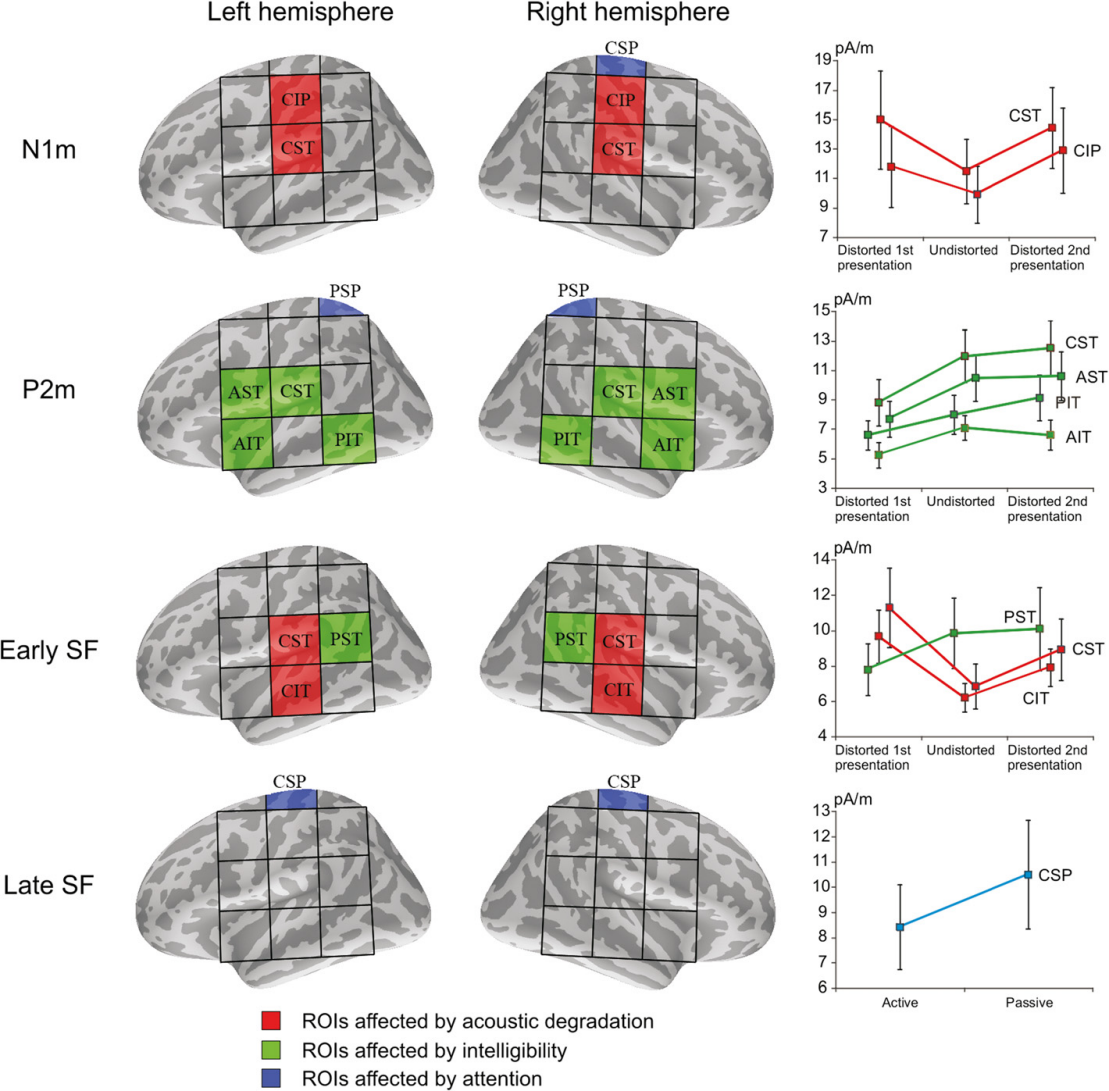
**Summary of the MNE results. Left**: Regions of interest (ROIs) in the left and right hemisphere modulated by the acoustic quality of speech, the intelligibility of speech, and the attentive state of the listener during the N1m, P2m, Early SF and Late SF time ranges. **Right**: MNE source currents within the affected ROIs during each time range. During the N1m time range, sensitivity to acoustic structure was evident in the central superior temporal (CST) and central inferior parietal (CIP) areas where stimulus degradation increased cortical activation. During the P2m time range, areas surrounding the CST in the superior and inferior temporal areas were more active during intelligible speech, regardless of whether the speech was acoustically distorted or not. In the early SF time range, the central temporal areas (CST & CIT) exhibited stronger activity to acoustically degraded speech while the posterior superior temporal (PST) area was sensitive to the intelligibility of speech. During the late SF, an attention-related effect was observable in the parietal brain areas (CSP). Error bars indicate the standard error of the mean (SEM).

In the second stage of the analyses, we elucidated how activity in auditory cortex and the surrounding areas evolves over time during the processing of connected speech. To this end, brain activity was analyzed using minimum norm estimates (MNEs) during the time ranges of transient (Figure 6) and sustained (Figure 7) brain activity. The current distribution was studied by dividing the temporal and parietal cortical surface into 24 regions of interest (ROIs) and calculating the mean currents over the voxels inside these regions (see Figure 2). The statistical results on the intensity of cortical activity within specific ROIs are given in Table 1. A graphical summary of the effects of acoustic degradation and intelligibility of speech on the strength of cortical activity within specific ROIs is shown in Figure 8.

**Table 1:** Statistical analyses of the MNE data

**Effect**	***F***	**df1**	**df2**	***p***
**N1m time range**				
*Hemisphere*				
Anterior inferior parietal	19,81	1	7	<0.01
Anterior superior temporal	9,83	1	7	<0.02
Posterior superior parietal	7,78	1	7	<0.05
*Degradation*				
Central inferior parietal	4,17	2	14	<0.05
Central superior temporal	5,62	2	14	<0.02
*Hemisphere × Attention*				
Central superior parietal	7,05	1	7	<0.05
**P2m time range**				
*Hemisphere*				
Anterior superior temporal	7,78	1	7	<0.05
Central inferior temporal	6,44	1	7	<0.05
Posterior superior parietal	9,37	1	7	<0.02
*Attention*				
Posterior superior parietal	5,76	1	7	<0.05
*Attention × Degradation*				
Anterior superior temporal	6,01	2	14	<0.02
Central superior temporal	5,48	2	14	<0.02
Anterior inferior temporal	4,77	2	14	<0.05
Posterior inferior temporal	4,61	2	14	<0.05
**Early SF time range**				
*Degradation*				
Central superior temporal	5,28	2	14	<0.02
Central inferior temporal	3,89	2	14	<0.05
*Attention × Degradation*				
Central inferior parietal	4,44	2	14	<0.05
Posterior superior temporal	4,20	2	14	<0.05
**Late SF time range**				
*Hemisphere*				
Central superior parietal	10,05	1	7	<0.02
Posterior superior parietal	11,33	1	7	<0.02
*Attention*				
Central superior parietal	6,84	1	7	<0.05
*Attention × Degradation*				
Central inferior parietal	4,10	2	14	<0.05

ANOVA results for the hemisphere, degradation and attention of the source current strength within specific ROIs during the N1m, P2m, early SF and late SF time windows. Only statistically significant effects are shown.

#### N1m time range

The strongest source currents during the N1m time range were localized in the vicinity of the auditory cortical areas (Figure 6). Cortical activity was more pronounced in the right hemisphere (11-12 pA/m) than in the left (7-9 pA/m) within the anterior inferior parietal (AIP), anterior superior temporal (AST) and posterior superior parietal (PSP) regions. Furthermore, the mean current strength was also higher in the right hemisphere than in the left within the central superior parietal (CSP) area, although this hemispheric asymmetry was observed only during passive listening of the sentences. The mean currents during the N1m were affected by stimulus degradation in the central inferior parietal (CIP) and central superior temporal (CST) regions. In the case of CIP, post-hoc tests indicated that the mean current was higher during the second presentation of the degraded sentences (13 pA/m) than during the undistorted sentences (10 pA/m; *p*<0.05). Within the CST, both the first and the second presentation of degraded speech yielded stronger activation (14 pA/m and 15 pA/m) than undistorted speech (12 pA/m; *p*<0.02). Altogether, the central parts of the superior temporal and inferior parietal regions were responsive to acoustic degradation of speech during the N1m time range.

#### P2m time range

The current distribution was more widespread during the P2m than during the N1m (Figure 6). The mean currents were stronger in the right-hemispheric AST, PSP and central inferior temporal (CIT) regions (10-12 pA/m) than in their left-hemispheric counterparts (7-9 pA/m). Moreover, the average current strength was higher during passive (11 pA/m) than active (9 pA/m) listening within the PSP. A number of regions exhibited sensitivity to the intelligibility of speech in the passive listening condition: In the AST and CST, the cortical activity was weaker during the (unintelligible) first presentation of the degraded sentences (7-9 pA/m) than during the undistorted stimuli (10-12 pA/m) and the second presentation of the distorted stimuli (11-13 pA/m; post-hoc *p*<0.05, except CST unintelligible vs. undistorted: *p*<0.058). A comparable intelligibility effect was observed also in the anterior inferior temporal (AIT) and posterior inferior temporal (PIT) areas, with the unintelligible stimuli yielding weaker currents than the intelligible ones. In the PIT, however, only the second presentation of degraded speech (9 pA/m) yielded significantly stronger currents than the first presentation of the same stimuli (6 pA/m; *p*<0.05). In turn, in the AIT, only the undistorted sentences (7 pA/m) resulted in significantly stronger activation than the first presentation of degraded stimuli (5 pA/m; *p*<0.05). Taken together, these findings suggest that a number of regions within the temporal cortex could be sensitive to the intelligibility of speech during the P2m time window, given that increased currents seem to be associated with the presentation of intelligible sentences.

#### Early SF time range

Cortical activation during the sustained field (SF) was distributed over a large area within the temporal and parietal regions (Figure 7). As demonstrated in Figure 8, the CST and CIT regions were sensitive to degradation of speech during the early SF, with the distorted sentences resulting in stronger activity (10-11 pA/m) than the undistorted ones (7-8 pA/m). Furthermore, the effect of acoustic degradation on the early SF was dependent on attention within the CIP and PST areas. The PST, in particular, was sensitive to speech intelligibility in the passive condition as the unintelligible first presentation of degraded speech (7 pA/m) yielded a weaker mean current than in the two other conditions (9-10 pA/m; *p*<0.05). A similar effect was observed also in the CIP, although it failed to reach statistical significance in the post-hoc comparisons. Thus, it seems that PST and, possibly, CIP are sensitive to speech intelligibility during the early part of the SF.

#### Late SF time range

As shown in Figure 7, activation within the CSP and PSP was stronger in the right hemisphere (10-12 pA/m) than in the left (8-10 pA/m) during the late SF. In addition, mean currents were higher in the CSP during passive (11 pA/m) than active (8 pA/m) listening of speech. The current strength during the late SF varied also with stimulus degradation in the CIP, although this effect depended on the listener’s attentional state. More specifically, in the passive condition, the first presentation of the degraded sentences resulted in weaker currents (7 pA/m) than during the second presentation of the degraded stimuli (11 pA/m; *p*<0.06). The mean current during the undistorted sentences (9 pA/m) was also stronger than the current elicited by the first presentation of the degraded sentences, although the difference was not statistically significant.

## Discussion

In the current study, we set out to investigate how the intelligibility of connected speech is reflected in behavioral measures as well as in the concomitant activity in the auditory cortex and surrounding brain areas. By varying the intelligibility of the stimuli while keeping the acoustic features of the stimuli constant, the experimental design allowed us to tentatively identify cortical processing related to speech comprehension. Initially unintelligible, acoustically distorted sentences resulting in a 30% subjective intelligibility rating, were perceptually changed by presenting intact, undistorted versions of the sentences. Upon a second presentation of the acoustically distorted versions of the sentences, their intelligibility increased markedly, up to 80%. These perceptual changes were reflected in the transient and sustained activation of auditory cortex and surrounding brain areas.

In the gradiometer analyses, local activity of the auditory cortex at 100 ms as indexed by the N1m response was sensitive to the acoustic structure of speech in that the distorted stimuli elicited stronger activation with an earlier peak latency than the undistorted stimuli. An increase of response amplitude and decrease of latency was observed also at around 200 ms, in the P2m response. The amplitude and latency effects of the P2m were substantially more pronounced in the right hemisphere than in the left. These findings indicate that transient activity of the auditory cortex is sensitive to the acoustic properties of sound during the early (up to 300 ms) processing stages of connected speech, and that the right hemisphere is more sensitive to acoustic variability than the left. The initial transient responses were followed by a sustained response, arising at around 300 ms, and appearing to consist of an early (300-1000 ms) and a late (1000-3000 ms) phase. The early phase was more prominent in the left hemisphere and increased in amplitude when subjects attended to the stimuli. Compared to the preceding transient activity, the sustained activation was less sensitive to acoustic distortion of speech.

In the MNE analyses, the auditory cortex and surrounding areas exhibited divergent, bilateral activity patterns associated with acoustic feature processing and speech intelligibility. During the N1m time range, an increase in cortical activity due to stimulus degradation was observed in regions extending from the superior temporal gyrus (auditory cortex; CST in the current notation) to the inferior parts of the postcentral gyrus (CIP). Interestingly, a number of areas within the temporal cortex were sensitive to speech intelligibility during the P2m time range, with the intelligible stimuli - both distorted and undistorted - resulting in stronger activity than the unintelligible stimuli. This activation encompassed the auditory cortex, the inferior frontal gyri (including Broca’s area; AST), the anterior part of the superior temporal gyrus (AIT), and the posterior part of the inferior temporal gyrus (PIT). During the early phase of the SF (300-1000 ms), the auditory cortex was more active in response to the distorted than the undistorted sentences, regardless of their intelligibility. In contrast, cortical activity in the posterior parts of the superior temporal gyrus (including Wernicke’s area; PST) was stronger only during intelligible speech, regardless of whether the stimulus material was acoustically intact or distorted.

In the present experiment, the stimuli were distorted by using amplitude quantization, which has been shown to decrease substantially the intelligibility of isolated speech sounds (see, e.g. [18,43]). This was also the case in the current study, as the distorted sentences were initially very difficult to understand. However, after the subject was exposed to the undistorted versions of the sentences, the comprehensibility of the distorted sentences increased considerably. It is unlikely that the intelligibility effect seen in both behavioral and brain measures is an effect due solely to the repetition of the distorted stimuli given that the gap between repetition (i.e., between Session 1 and 3) was around 20 minutes. This time span makes it improbable that the subject could have been drawing on any echoic or short-term memory resources. Instead, this increase in comprehension was most likely caused by top-down mechanisms utilizing the long-term memory representations which were instantly activated (or primed; e.g. [44]) during listening to the intact versions of the stimuli. Similar changes in the perception of acoustically identical speech-like stimuli have been observed also using noise-vocoded sentences [5,22] and sine-wave speech stimuli [45]. However, in these cases the perceptual changes were brought about through extended training sessions, whereas in the current context, these effects were immediate, and observable after already a single presentation of the undistorted versions of the stimuli. Thus, depending on the experimental setup, it now appears to be possible to study brain mechanisms of perceptual learning occurring over a long time scale as well as rapid activation of linguistic memory representations.

The changes in the acoustic structure of the speech stimuli brought about by distortion were reflected in both the transient and sustained activation patterns of the auditory areas. In contrast, the temporal regions anterior and posterior to auditory cortex (area CST) were insensitive to degradation. The observed increase in the amplitude of the transient responses is in line with earlier results employing the same distortion method [17-19]. These studies have demonstrated that the amplitude increase of the N1m and P2m responses is related to an increase in harmonic frequencies in the signal spectrum brought about by quantization. According to this explanation, the additional harmonics activate a larger number of neurons involved in the pitch extraction process. In the current study the latency of the transient responses was also affected by the distortion, with earlier N1m and P2m latencies for the distorted sentences. This finding deviates from our earlier results using isolated speech sounds (~200 ms vowel sounds), for which the response latencies remained unchanged when the stimuli were distorted. One reason for these differences may lie in the experimental design: in previous studies by Miettinen et al. [17-19], short-duration isolated vowels were repeated at a fast rate whereas in the current case long-duration sentences with a complex, continually evolving spectral structure were presented with intervening long silent periods. Similar latency results were recently reported by Obleser and Kotz [46], who found that the N1m response peaks earlier and is larger in amplitude for distorted sentences than for their undistorted counterparts.

In the present experiment, the auditory cortex was highly responsive to distortion of speech, which is consistent with prior hemodynamic studies showing that the core auditory areas are sensitive to acoustic differences in speech stimuli [21-28]. The regions surrounding the auditory cortex, in turn, were sensitive to the intelligibility of speech, with stronger activation elicited by intelligible speech regardless of whether the stimulus material was distorted. These findings are congruent with the above fMRI results, in particular with those by Okada et al. [25], who observed a bilateral sensitivity of both the anterior and posterior superior temporal regions to speech intelligibility. Importantly, we observed that, already during the P2m time range, areas in the vicinity of the auditory cortex were sensitive to speech intelligibility as well (see Figure 8). This intelligibility effect, observable presumably because of the temporal resolution of the MEG, might reflect the influence of top-down feedback from higher-order cortical areas on the activity of auditory cortex. Similar findings have also been reported by Wild et al. [30] and Sohoglu et al. [47], who demonstrated that prior expectations of speech content modulate the activity of auditory cortex during listening to distorted speech.

The novel experimental paradigm introduced here points to several interesting possibilities for future research. Firstly, one should keep in mind that the current intelligibility effects in cortical activity were observed in the passive condition which always followed the active condition, and it therefore remains to be clarified whether there were carry-over effects from one to the other. This interesting issue, related to the decay time of recognition memory, clearly deserves further study. Secondly, an important question for future investigation is how the number of sentences used in the experiment affects intelligibility and behavioral performance. Assuming the memory system probed with the current paradigm has a capacity limitation, increasing the number of sentences should at some point lead to decreased performance. Indeed, the intelligibility of the sentences in the current study might have been facilitated by the limited number of words and sentence stubs used to construct the stimulus material. Thirdly, in studying the priming of memory representations of speech, a further step, requiring a larger set of sentences than in the current case, would be to average brain responses selectively based on the behavioral performance (in terms of unintelligible vs. intelligible sentences), and to study how this is reflected in the activation of brain areas. We expect that this approach would lead to even more pronounced intelligibility effects in cortical activity than those reported here.

## Conclusions

The current study utilized an experimental setting which allowed for physically identical, distorted speech stimuli to be perceived as either unintelligible or intelligible due to a single intervening exposure to the undistorted versions of the stimuli. In the N1m time range (~100 ms), the auditory areas within the superior temporal sulcus seem to be sensitive to *acoustic degradation* of speech. Thereafter, in the time range of P2m (200-300 ms), auditory cortex as well as cortical regions anterior and posterior to this area appear to be responsive to the *intelligibility* of speech. Following this transient brain activity, during the early SF (at 300-1000 ms), the region most sensitive to speech intelligibility was located in the posterior part of the superior temporal gyrus of each hemisphere. In terms of auditory scene analysis [1], the current experimental setup could be seen as providing a new methodological approach to studies of auditory scene analysis. This phenomenon has traditionally been studied using sequencing of auditory stimuli alternating in, for example, frequency, intensity, or spatial location (see, e.g. [48]), whereas the experimental distorted-undistorted-distorted setup proposed here allows one to study how meaningful entities such as speech are segregated from a noisy signal by matching incoming acoustic signals to memory representations. Our results indicate that in building a coherent whole from various auditory confluences, the acoustic attributes of incoming auditory signals are identified rapidly in the auditory cortex within the first 100 ms. The activity of the auditory cortex appears to be modulated through feedback connections, as indicated by the fact that already at 200 ms sensitivity to speech intelligibility emerges in this and surrounding areas. In making the unintelligible suddenly intelligible, these modulations can result in substantial changes in the way we perceive connected speech.

## Abbreviations

AEF: Auditory evoked field; ANOVA: Analysis of variance; ECD: Equivalent current dipole; EEG: Electroencephalography; fMRI: Functional magnetic resonance imaging; HPI: Head-position indicator; MEG: Magnetoencephalography; MNE: Minimum norm estimate; ROI: Region of interest; SF: Sustained field; sLORETA: Standardized low resolution brain electromagnetic tomography; SNR: Signal-to-noise ratio; USQ: Uniform scalar quantization.

## Competing interests

The authors declare that they have no financial or any other competing interests.

## Authors’ contributions

HT, PM and PA designed the experimental setup of the study and prepared the auditory stimuli. IM carried out data acquisition, performed the statistical analyses and prepared the first draft of the manuscript. All authors participated in the writing process and have approved the final version of the manuscript.
